# Lymphocyte phenotype during severe sepsis and septic shock

**DOI:** 10.1186/2197-425X-3-S1-A622

**Published:** 2015-10-01

**Authors:** M Le Dorze, V Tarazona, C Brumpt, H Moins-Teisserenc, AC Lukaszewicz, D Payen

**Affiliations:** Department of Anesthesiology and Critical Care Lariboisiere Hospital, UMR INSERM 1160, University Paris 7, Paris, France; Department of Anesthesiology and Critical Care Lariboisiere Hospital, UMR INSERM 1160, University Paris, Paris, France; Hematology Lariboisiere Hospital, University Paris 7, Paris, France; Immunology Saint-Louis Hospital, University Paris 7, Paris, France

## Introduction

Two major immuno-inflammatory phases are well documented during sepsis: an early active inflammatory phase regulated followed by a deactivated inflammatory phase leading to immunosuppression. Innate immunity, such as monocyte HLA-DR expression (mHLA-DR), has been shown constantly and rapidly decreased. Adaptive immunity modifications have been reported, this appeared less documented, particularly in comparison with healthy volunteers which have been rarely reported.

## Objectives

The aim of this study is 1- to describe lymphocyte phenotypes in healthy volunteers 2- to study the phenotype in early (< D7) and late (>D7) phases of severe sepsis 3- to test the relation between lymphocyte phenotypes and prognosis.

## Methods

Monocenter study on patients with severe sepsis and septic shock. Data recorded: demographic, clinical severity, blood cell count, mHLA-DR, lymphocyte phenotype (flow cytometry, CD3+, CD3+CD4+, CD3+CD8+, CD19+, CD16+CD56+). Samples taken as part of routine care without additional blood collection and did not required patient´s consent. Patients data compared with a historical group of healthy volunteers from the laboratory. Statistics: results expressed as median (25^th^-75^th^ percentile), Mann-Whitney test, Pearson correlation.

## Results

45 septic patients (9 severe sepsis, 36 septic shock), age 66 (52-85), sex (29M/16F), origin of sepsis (pulmonary 30%, abdominal 35%, others 30%), SAPS2 at admission 51 (40-63), SOFA score (day of phenotype) 9 (5-13), overall mortality at day 7: 22% and day 28: 48%, delay between the inflammatory insult and lymphocyte phenotype was 2 days (1-10). 48 healthy volunteers in the control group: age 42 (31-55), sex (24M/24F). The overall lymphopenia in septic patients was -37% compared to controls, mainly due to CD4 T lymphocytes depletion (Table). This T lymphopenia was more marked in the early phase (< 7D) than in the late phase (>7D) (p = 0.006). Early lymphocyte phenotype was not associated with mortality at day 7; as was not late lymphocyte phenotype with mortality at day 28. There was a correlation between lymphopenia and mHLA-DR (r0.55, p0.00009). There was no correlation between lymphocyte phenotype and severity scores.

**Table 1:** Table 1

	Control group (n=48)	Sepsis group (n=45)	p
**Lymphocytes (n/mm3)**	1586 (1372-1991)	1000 (660-1495)	0.12
**T Lymphocytes CD3+**	1182 (949-1524)	609 (258-1038)	< 0.05*
**CD3+CD4+**	685 (595-1035)	378 (156-668)	< 0.05*
**CD3+CD8+**	431 (301-508)	200 (90-338)	0.33
**B Lymphocytes CD19+**	143 (96-338)	149 (75-252)	0.14
**NK CD16+CD56+**	161 (114-268)	161 (114-268)	0.83
**mHLA-DR (AB/C)**	39970 (35480-46480)	5700 (3545-10072)	< 0.05*

*[Lymphocyte Phenotype]*.

## Conclusions

In severe sepsis including shock, lymphopenia was predominant on CD4+ T lymphocytes, mainly at the early phase, in association with decreased expression of mHLA-DR, suggesting synergistic changes in innate and adaptive immunity. Such monitoring for innate and adaptive immunity may help to decide the suitable drug to boost immunity, such as IFNγ or IL-7.
